# Supplementary material for: Socioeconomic determinants of Schistosoma mansoni infection using multiple correspondence analysis among rural western Kenyan communities: Evidence from a household-based study
Source: PLoS One. 2021 Jun 23;16(6):e0253041. doi: 10.1371/journal.pone.0253041 (PMC8221481; doi:10.1371/journal.pone.0253041)
Supplement: Attachment — Submitted filename: PONE-D-20-05468_reviewer.pdf [file pone.0253041.s003.pdf]

PLOS ONE

Socioeconomic determinants of schistosomiasis infection using multiple correspondence analysis among rural western Kenyan communities: evidence from a schistosomiasis household-based research.

--Manuscript Draft--

**Manuscript Number:**	PONE-D-20-05468
**Article Type:**	Research Article
**Full Title:**	Socioeconomic determinants of schistosomiasis infection using multiple correspondence analysis among rural western Kenyan communities: evidence from a schistosomiasis household-based research.
**Short Title:**	Socioeconomic determinants of schistosomiasis infection using multiple correspondence analysis
**Corresponding Author:**	ISAIAH OMONDI<br>Kenya Medical Research Institute<br>KISUMU, KENYA
**Keywords:**	Schistosomiasis, Rural environment, Social economic status, Multiple Correspondence Analysis, Western Kenya
**Abstract:**	Socioeconomic inequality including wealth distribution is a barrier to implementation of health policies. Although wealth distribution can be measured eectively using household data on durable assets, such data is rarely analysed in schistosomiasis related studies using Socio-economic Status (SES) analysis tools such as Multiple Correspondence Analysis (MCA), which can create more reliable wealth quintiles. We therefore evaluated socioeconomic determinants of schistosomiasis using MCA on household data among adult population in western Kenya. Twelve villages, 6 villages that had showed the greatest decrease in Schistosoma mansoni prevalence and 6 villages that showed relatively lower decrease over the years, based on the previous Schistosomiasis Consortium for Operational Research and Elimination (SCORE) report from western Kenya were purposively included into the study. One stool sample from 300 randomly selected adults from each stratum were examined based on four slides for Schistosoma mansoni eggs using Kato Katz technique and MCA implemented on the household assets. A log binomial regression model was used to identify the inuence of age, household size, wealth class, occupation, education status, main water supply, gender and sub-county of residence on schistosomiasis infection; parasite densities were then compared across variables that were signicant based on multivariate log binomial model analysis using a mixed model. Overall prevalence of schistosomiasis was 41.3%. Only sex and household size were signicant predictors in the multivariate model. Males were associated with signicantly higher prevalence compared to the females (aPR = 1.37; 95% CI = 1.141.66). In addition, households with at least 4 persons had higher prevalence compared to those with less than 4 (aPR = 1.29; 95% CI = 1.031.61). However, there was no dierence in prevalence between the wealth classes. Socioeconomic status based on wealth index is not a contributing factor to schistosomiasis prevalence. Keywords Schistosomiasis, Rural environment, Social economic status, Multiple Correspondence Analysis, Western Kenya
**Order of Authors:**	ISAIAH OMONDI
	Maurice R. Odiere
	Fredrick Rawago
	Pauline N. Mwinzi
	Carl H Campbell
	Rosemary Musuva
**Additional Information:**	
**Question**	**Response**

Powered by Editorial Manager® and ProduXion Manager® from Aries Systems Corporation

**Financial Disclosure**<br><br>Enter a financial disclosure statement that describes the sources of funding for the work included in this submission. Review the submission guidelines for detailed requirements. View published research articles from *PLOS ONE* for specific examples.<br><br>This statement is required for submission and **will appear in the published article** if the submission is accepted. Please make sure it is accurate.<br><br>**Unfunded studies**<br>Enter: *The author(s) received no specific funding for this work.*<br><br>**Funded studies**<br>Enter a statement with the following details:<br>• Initials of the authors who received each award<br>• Grant numbers awarded to each author<br>• The full name of each funder<br>• URL of each funder website<br>• Did the sponsors or funders play any role in the study design, data collection and analysis, decision to publish, or preparation of the manuscript?<br>• **NO** - Include this sentence at the end of your statement: *The funders had no role in study design, data collection and analysis, decision to publish, or preparation of the manuscript.*<br>• **YES** - Specify the role(s) played.<br><br>* typeset	This study received financial support from the university of Georgia Research foundation, inc. which was funded by the Bill and Melinda Gates Foundation for the SCORE Project. The funders had no role in the study design, data collection and analysis, decision to publish or preparation of the manuscript
**Competing Interests**<br><br>Use the instructions below to enter a competing interest statement for this submission. On behalf of all authors, disclose any competing interests that could be perceived to bias this work—acknowledging all financial support and any other relevant financial or non-financial competing interests.<br><br>This statement **will appear in the published article** if the submission is accepted. Please make sure it is	The authors have declared that no competing interests exist.

accurate. View published research articles from *PLOS ONE* for specific examples.<br><br>**NO authors have competing interests**<br><br>Enter: *The authors have declared that no competing interests exist.*<br><br>**Authors with competing interests**<br><br>Enter competing interest details beginning with this statement:<br><br>*I have read the journal's policy and the authors of this manuscript have the following competing interests: [insert competing interests here]*<br><br>* typeset	
**Ethics Statement**<br><br>Enter an ethics statement for this submission. This statement is required if the study involved:<br><br>• Human participants<br>• Human specimens or tissue<br>• Vertebrate animals or cephalopods<br>• Vertebrate embryos or tissues<br>• Field research<br><br>Write "N/A" if the submission does not require an ethics statement.<br><br>General guidance is provided below. Consult the submission guidelines for detailed instructions. **Make sure that all information entered here is included in the Methods section of the manuscript.**	The study protocol was reviewed and approved by KEMRI's Scientific and Ethics Review Unit (SERU) KEMRI SSC no. 3267. Thereafter, permission was obtained from the County administration, Ministry of Education and Ministry of Health. The purpose of the study and its objectives were explained to the local authorities, pre-school and sprimary school teachers, CHEWs and opinion leaders. Written Informed consent was obtained from the participating respondents. Subjects were assured about confidentiality of information obtained from them and personal identifiers were removed from the data set before analysis.

Powered by Editorial Manager® and ProduXion Manager® from Aries Systems Corporation

**Format for specific study types**<br><br>**Human Subject Research (involving human participants and/or tissue)**<br>• Give the name of the institutional review board or ethics committee that approved the study<br>• Include the approval number and/or a statement indicating approval of this research<br>• Indicate the form of consent obtained (written/oral) or the reason that consent was not obtained (e.g. the data were analyzed anonymously)<br><br>**Animal Research (involving vertebrate animals, embryos or tissues)**<br>• Provide the name of the Institutional Animal Care and Use Committee (IACUC) or other relevant ethics board that reviewed the study protocol, and indicate whether they approved this research or granted a formal waiver of ethical approval<br>• Include an approval number if one was obtained<br>• If the study involved *non-human primates*, add *additional details* about animal welfare and steps taken to ameliorate suffering<br>• If anesthesia, euthanasia, or any kind of animal sacrifice is part of the study, include briefly which substances and/or methods were applied<br><br>**Field Research**<br><br>Include the following details if this study involves the collection of plant, animal, or other materials from a natural setting:<br>• Field permit number<br>• Name of the institution or relevant body that granted permission	
**Data Availability**<br><br>Authors are required to make all data underlying the findings described fully available, without restriction, and from the time of publication. PLOS allows rare exceptions to address legal and ethical concerns. See the PLOS Data Policy and FAQ for detailed information.	No - some restrictions will apply

Powered by Editorial Manager® and ProduXion Manager® from Aries Systems Corporation

A Data Availability Statement describing where the data can be found is required at submission. Your answers to this question constitute the Data Availability Statement and **will be published in the article**, if accepted.<br><br>**Important:** Stating 'data available on request from the author' is not sufficient. If your data are only available upon request, select 'No' for the first question and explain your exceptional situation in the text box.<br><br>Do the authors confirm that all data underlying the findings described in their manuscript are fully available without restriction?	
**Describe where the data may be found in full sentences. If you are copying our sample text, replace any instances of XXX with the appropriate details.**<br><br>• If the data are **held or will be held in a public repository**, include URLs, accession numbers or DOIs. If this information will only be available after acceptance, indicate this by ticking the box below. For example: *All XXX files are available from the XXX database (accession number(s) XXX, XXX.*).<br>• If the data are all contained **within the manuscript and/or Supporting Information files**, enter the following: *All relevant data are within the manuscript and its Supporting Information files.*<br>• If neither of these applies but you are able to provide **details of access elsewhere**, with or without limitations, please do so. For example:<br><br>*Data cannot be shared publicly because of [XXX]. Data are available from the XXX Institutional Data Access / Ethics Committee (contact via XXX) for researchers who meet the criteria for access to confidential data.*<br><br>*The data underlying the results presented in the study are available from (include the name of the third party*	Data cannot be shared publicly because Kenya Medical Research Institute Data Privacy policies. Data are available from the Kenya Medical Research Institute Institutional Data Access for researchers who meet the criteria for access to confidential data.

Powered by Editorial Manager® and ProduXion Manager® from Aries Systems Corporation

and contact information or URL).<br>• This text is appropriate if the data are owned by a third party and authors do not have permission to share the data.<br><br>* typeset	
Additional data availability information:	

Manuscript

Click here to access/download;Manuscript;manuscript_22mar2020.pdf

Socioeconomic determinants of schistosomiasis infection using multiple correspondence analysis among rural western Kenyan communities: evidence from a schistosomiasis household-based research.

Isaiah Omondi[1], Maurice R. Odiere[1], Fredrick Rawago[1], Pauline N. Mwinzi[1], Carl Campbell[2], Rosemary Musuva[1]

[1] *Neglected Tropical Diseases Branch, Center for Global Health Research, Kenya Medical Research Institute (KEMRI), Kisumu, Kenya.*
[2] *Center for Tropical and Emerging Global Diseases and Department of Microbiology, University of Georgia, Athens, Georgia, United States of America.*

Abstract

Socioeconomic inequality including wealth distribution is a barrier to implementation of health policies. Although wealth distribution can be measured effectively using household data on durable assets, such data is rarely analysed in schistosomiasis related studies using Socio-economic Status (SES) analysis tools such as Multiple Correspondence Analysis (MCA), which can create more reliable wealth quintiles. We therefore evaluated socioeconomic determinants of schistosomiasis using MCA on household data among adult population in western Kenya. Twelve villages, 6 villages that had showed the greatest decrease in *Schistosoma mansoni* prevalence and 6 villages that showed relatively lower decrease over the years, based on the previous Schistosomiasis Consortium for Operational Research and Elimination (SCORE) report from western Kenya were purposively included into the study. One stool sample from 300 randomly selected adults from each stratum were examined based on four slides for *Schistosoma mansoni* eggs using Kato Katz technique and MCA implemented on the household assets. A log binomial regression model was used to identify the influence of age, household size, wealth class, occupation, education status, main water supply, gender and sub-county of residence on schistosomiasis infection; parasite densities were then compared across variables that were significant based on multivariate log binomial model analysis using a mixed model. Overall prevalence of schistosomiasis was 41.3%. Only sex and household size were significant predictors in the multivariate model. Males were associated with significantly higher prevalence compared to the females (aPR = 1.37; 95% CI = 1.14–1.66). In addition, households with at least 4 persons had higher prevalence compared to those with less than 4 (aPR = 1.29; 95% CI = 1.03–1.61). However, there was no difference in prevalence between the wealth classes. Socioeconomic status based on wealth index is not a contributing factor to schistosomiasis prevalence.

Keywords

Schistosomiasis, Rural environment, Social economic status, Multiple Correspondence Analysis, Western Kenya

Introduction

Schistosomiasis affects an aproximately 207 million people globally [1] and this accounts for close to 1.9 million disability-adjusted life years (DALYs) annually [2]. Of the overall global cases, 93% (192 million) are reported within Sub-Saharan Africa region [3] whereby the disease is prevalent especially among poor communities without access to safe drinking water and adequate sanitation [4]. A higher percentage (70%) of those at risk of schistosomiasis infection is estimated to be school going children aged between 5 to 14 years [5]. Although schistosomiasis is regarded as a social disease, studies on the association of socioeconomic conditions with schistosomiasis have been equivocal, with some indicating association, as others indicate no effect. A cross-sectional study in Walukuba Division bordering Lake Victoria, Jinja District found out that wealth index is a significant risk factor for infection with *schistosoma mansoni* [6]. Another study in North Western Tanzania that used Principal Component Analysis (PCA) to create wealth quintiles demonstrated that control interventions against schistosomiasis and intestinal worms contributed towards improvement in socio-economic status of a community population [7]. Other studies have also shown that persistent schistosomiasis prevalence among school children could be attributed to socioeconomic inequality [8]. In Kenya the role of socioeconomic factors in schistosomiasis transmission has been examined by several studies. For example, a cross sectional study involving school children from eight different primary schools in Mbita district found no effect of socioeconomic factors on *Schistosoma mansoni* infection risk [9]. Therefore, additional studies on the influence of socioeconomic factors are warranted, especially in high transmission settings such as western Kenya that need an adaptable schistosomiasis control strategy that considers local differences in disease ecology. Standard Socioeconomic Status (SES) measures employ monetary information, including income or consumption expenditures. This however poses a caveat since the collection of accurate income data calls for extensive resources for household surveys; for instance, provisions need to be made for households and individuals drawing income from multiple sources. Although consumption or expenditure measures are much more reliable and are easier to collect than income, it requires extensive data collection which could be time-consuming and therefore extra cost implications. Due to resource constraints when measuring household income or expenditure in low- and middle-income country settings, adoption of other SES evaluation methods that could streamline variable requirement has been necessitated hence allowing rapid data collection. Besides income or expenditure, data are collected for variables that capture living standards, for example household ownership of durable assets (e.g. Cupboards, motorvehicle) and infrastructure and housing characteristics (e.g. source of water, sanitation facility). The use of asset-based indices of evaluating wealth in developing countries-instead of per capita expenditures has gained popularity in the past few years especially in instances where data on expenditures are inaccessible or too costly to obtain. Traditional methods of creating SES indices have proved to suffer from recall bias, sea-

sonality and data collection time. When per capita expenditure data are missing, the use of an asset index can clearly provide useful guidance to the order of magnitude of rich-poor-differentials [10]. There exists a vast volume of studies that have recommended asset-based methods of creating SES index which tend to be superior over traditional methods which rely on income and consumption expenditures. Such methodologies include the Filmer-Pritchett Principal Component Analysis (PCA), polychoric PCA and Multiple Correspondence Analysis (MCA). PCA is computationally easier, uses data that can easily be obtained from household surveys and uses all the asset variables in reducing the dimensionality of the data and therefore is a valid method for describing SES differentiation within a population [11]. Filmer-Pritchett procedure for conducting PCA requires that the variable be divided into a set of dummy indicators [12]. However, this could lead to deterioration of performance and probably produce a model with lower explanatory power. Therefore, polychoric correlations PCA method tend to outperform Filmer-Pritchett PCA if the proportion of explained variance is of importance [14]. A study comparing the performance of Filmer-Pritchett PCA, Polychoric PCA and MCA found that although the results were identical, MCA accounted for a higher proportion of the total variance of the household assets variables as compared to the other two methods [15]. The MCA model is also able to take into account variables measured on quantitative scale which is not possible with ordinary and polychoric PCA [16]. However, studies employing MCA to investigate socioeconomic disparities about schistosomiasis are still lacking. Health inequality data, including *Schistosoma Mansoni* prevalence are often collected in community-based surveys but rarely analysed from a socioeconomic point of view. Yet, such data if properly analysed would be necessary for monitoring health inequalities and the impact of schistosomiasis control interventions at the microeconomic level. The aim of this study was to establish the relationship between household SES and inequalities ~~in~~ schistosomiasis prevalence in a schistosomiasis endemic area of rural western Kenya. The current study analyzed the relationship between socioeconomic status and risk of infection with schistosomiasis while controlling for measures of exposure to schistosomiasis such as water and sanitation facilities. This study hypothesized that the distribution of schistosomiasis within the western Kenya population is determined by socioeconomic factors.

Materials and methods

Study Area, economy and population

This community-based cross-sectional survey was conducted in the first quarter of 2018 in 12 villages in 3 sub-counties of Rarieda and Bondo (Siaya County) and Rachuonyo North sub-county (Homa bay County) in Western Kenya. Siaya county lies between latitude N 0°26′ to N 0°18′ and longitude E 33°58′ and W 34°33′ and shares Lake Victoria border towards the South with Homa Bay County. Both counties are in the *Schistosoma Mansoni* – endemic area of western Kenya and share many physical and socioeconomic environmental features. They include a tropical climate with Siaya experiencing average annual rainfall of 1572 mm and temperature of 21.7°C whereas Homabay experiences average annual rainfall of 1226 mm and temperatures of 22.5°C. The sub-counties border Lake Victoria

~~and~~ *Schistosoma mansoni* is highly endemic ~~in the area~~. The main socio-economic activities in the area are fishing, subsistence farming and petty trade. The main source of water for domestic use among the inhabitants of these areas is Lake Victoria which is the primary source of *Schistosoma mansoni* infection, ~~with an inverse association between proximity to the lake and *Schistosoma mansoni* infection prevalence [17].~~

Study Design

Villages for inclusion into this study were purposively selected from villages that participated in ~~our previous~~ Schistosomiasis Consortium for Operational Research and Evaluation (SCORE) studies. An example of such studies was a chemotherapy-based initiative that was undertaken to control schistosomiasis in ~~this~~ region that implemented school-based as well as village-wide annual mass drug administration (MDA) in varying treatment levels for 5 consecutive years (2011-2015) for 150 Kenyan villages situated on or near the eastern shore of Lake Victoria [19]. Six villages with the greatest decrease in *Schistosoma mansoni* prevalence (Responder villages) and other six where prevalence remained persistently high ('hot-spots') were selected for inclusion. Convenience sampling was then used to identify 50 adults from 50 households in each village. Informed consent obtained from study participants prior to enrolment into the study. Pre-designed and tested questionnaire was administered to the participants and a single stool and urine sample was collected for testing at the KEMRI laboratory.

Parasitological Assesment

Stool samples were collected from 600 participants. Each participant was provided with a plastic container for a stool sample and plastic cup for urine on the morning of the day of survey and instructed to bring the samples. Parasitological assessment was based on one stool sample (4 slides) per participant. Each slide was analyzed by the Kato-Katz technique for eggs of *Schistosoma mansoni*, and soil-transmitted helminths (*Ascaris lumbricoides*, *Trichuris trichiura* and hookworm). Approximately 41.7 mg of faeces was used for each slide. Eggs were counted and expressed in eggs per gram (EPG) by two independent microscopists and any discrepancy in results of the two was reconciled by comparing to results of a third independent and more experienced microscopist. World Health Organization (WHO) – proposed thresholds were used to classify the intensity of infection [21]. Individuals who tested positive for schistosomiasis were treated with praziquantel (PZQ), single dose of 40 mg/kg, whereas those who tested positive for soil-transmitted helminths were treated with a single dose of albendazole (400mg).

Collection of socioeconomic data

Socioeconomic data was collected from each participant who provided a stool sample for parasitological examination. Data on individual and household characteristics with a possible influence on schistosomiasis (gender, place of residence, age, source of water for domestic use, highest level of education attained, occupation of the household head, number of persons

in the household, type of latrine used by the household members was collected. In addition, information about factors indicating the family's financial situation (living in a brick or wood/mud house, existence of electricity and other assets such as refrigerator, sofa sets, dining table, car, tractor, cell phone, cupboard, wardrobe, motorcycle, bicycle) was collected through interviewing the household head using a structured interview with a questionnaire in English or if necessary in the local language, Dholuo. The questions were formulated according to standard questionnaires adjusted to local requirements and appropriateness. Data was captured real-time using a Commcare application installed in smart phones and submitted to a Central server on completion of each entry. Plausibility was thereafter checked, and data cleaned before the database was locked. All information on participants and their respective households was treated confidentially.

Multiple Correspondence Analysis

Multiple correspondence analysis (MCA) is a data analysis technique used to detect and represent underlying structures within several categorical dependent variables in a data set. It accomplishes this by representing data as points in a low-dimensional Euclidean space. The MCA procedure is could thus be regarded as the counterpart of principal component analysis for categorical data. [18] MCA could also be considered as an extension of simple correspondence analysis (CA) with regard to its applicability to a large set of categorical variables. MCA allows analysis of a set of observations construed by a set of both nominal and quantitative variables. To obtain wealth indices and their scores, the following socioeconomic indicator variables were considered: Materials used for walls of main house (no bricks, unburnt bricks, burnt bricks with mud, burnt bricks/stones with cement); material used for the floor of main house (not cemented, cemented, tiled); materials used for roofing of main house (thatch, iron sheets, tiles); source of water (no tap water, tap water outside house, tap water inside house); sanitation facility (none, pit latrine, ventilated improved pit latrine, flush toilet); household assets (radio, cupboard, bicycle, paddle dhow, telephone, television, motorcycle, car, engine dhow, sewing machine, refrigerator, wall clock, sofa) and existence of electricity. All the options above were transformed into binary/indicator variables prior to running the MCA model, with 1 denoting presence and 0 denoting absence of the respective facility/asset. The scores generated by the MCA model formed the basis for classifying individuals into five socio-economic quintiles with the first quintile as the poorest and the fifth quintile as the least poor. Finally, a binary variable was generated with the first three SES quintiles (i.e., poorest, second and third poorest) combined to form the 'poorest' category and the fourth and fifth quintiles combined to form the 'less-poor' category. The 'less-poor' category was then adopted as the reference category in the log-binomial and mixed regression models.

Statistical Analysis

The schistosomiasis data contained information on 600 individuals from the study area. This study aimed at exploring not only the prevalence of schistosomiasis but also its intensity. Shapiro-wilk test was employed to test the normality of the egg data and the results indicated a violation of the normality assumption, hence, a log-transform of the mean EPGs was

considered in order to attain normality. Several explanatory variables specific to the individuals, such as age, gender, education level, occupation, wealth index (based on MCA model), subcounty of residence, size of the household, main source of water and presence of latrine were considered in developing the final model. This study used univariate and multivariate regression models to examine the association between the prevalence of schistosomiasis and the socioeconomic indicators. Given that schistosomiasis is not a rare disease in Lake Victoria region, prevalence ratios as opposed to odds ratio (OR) were used to estimate risk of schistosomiasis infection. In addition, the prevalence ratios (PR) resulting from log-binomial model tend to be easier to interpret compared to odds ratio produced by logistic regression since log-binomial model uses a log link function whereas logistic regression model uses a logit link function [22]. In addition,compared to Mantel-Haenszel, Cox, Robust Poisson and logistic regression; log-binomial regression would be the best method for estimating prevalence ratios for intermediate prevalence [23], [24]. First, the univariate prevalence ratios for the demographic, socioeconomic and sanitation variables were generated by considering regression models with a single predictor variable. A generalized score statistic formed the basis for determining the overall significance of each variable, whereas the p-values and confidence intervals formed the basis for evaluating statistically significant differences between variable levels. The level considered to be least associated with the risk for contacting schistosomiasis was chosen as the reference category. To develop the multivariable model, several plausible interactions such as poverty and presence of latrine, poverty and highest education level, poverty and household size or poverty and the main source of water for domestic use were assesed, adding each interaction to the full model containing all the predictors presented in the univariate analysis. The results indicated that none of these interactions were significant and moreover the parameter estimates observed in the main effects indicated possible problems with multicollinearity probably resulting from model burdening correlations between the variables. The estimates produced for a small number of variables resulted in prevalence ratios counter-intuitive to the general understanding of the proposed risk factors. Among all the possible models, Schwarz Bayesian Criteria (SBC) was used to identify the best model since some studies have proven its superiority over other Information Criteria (AIC and BIC), Heuristic methods and Model diagnostics [25]. Those variables not entered into the multivariable model as significant predictors for schistosomiasis were assessed as confounders. After carrying out the univariate and multivariable analyses, we examined the intensity of infection by each of the predictors included in the final multivariate model. This analysis used the log-transformed geometric mean egg counts among those individuals infected with schistosomiasis. The geometric mean and confidence intervals for each level of the predictors included in the multivariable model were then calculated using a mixed model. This was achieved by modelling the log_{10} of the mean egg counts of each individual versus the covariates of interest, obtaining the least squares mean and confidence interval, and then transforming the estimates back to the original scale of the egg counts. An overall F-test of the mixed model was used to determine whether any differences in the mean log egg counts existed among the covariate levels. The pair-wise differences in means across all possible combinations of the covariate levels were calculated and assessed for significance using a Tukey multiple comparison adjustment [26]. Cases with missing stool lab results

were excluded from analysis. All analyses were completed in SAS (version 9.4).

Ethical Considerations

The review and approval of this study was done by the Scientific and Ethical Review Committee of the Kenya Medical Research Institute (KEMRI).

Results

Parasitological Results

Of the 600 study participants, 25.7% were males and 74.3% females. The population age ranged from 18 to 90 years (mean 44.7, SD 16.5, and median 42.5 years), with 21.5%, 56.5% and 22% aged below 30 years, between 30 to 60 years and above 60 years, respectively. The overall prevalence of schistosomiasis was 41.3% while the overall geometric mean egg count was 56.2 eggs per gram of feces. The prevalence of schistosomiasis among those aged below 30 years, between 30 to 60 years and above 60 years was 48.1%, 41.9% and 33.3% respectively while the prevalence among males and females was 53.3% and 37.2% respectively. In addition, arithmetic mean EPG peaked at 114.4 eggs per grams of feces among males and at 105.6 eggs per grams of feces for those aged below 30 years old.

Associations between infection and variables

~~Univariate analysis was used to model the probability of *S. mansoni* infection among levels of various potential risk factors, including demographic, socioeconomic and sanitation characteristics of the study participants. Among the demographic variables considered, there was a significant association between schistosomiasis infection and age group, gender and subcounty of residence~~. The most notable results were in the age group below < 30 years where prevalence of schistosomiasis was 1.44 (95% CI = 1.07 – 1.95) times the prevalence in those above > 60 years old as a reference group; males were associated with 1.43 (95% CI = 1.18 – 1.73) times the prevalence among females; whereas the prevalence among residents of Rachuonyo North sub-county was 1.51 (95% CI = 1.11 – 2.06) times the prevalence of those from Bondo sub-county (Table 1). Among the socio-economic

Table 1: **Prevalence ratios (PR) and 95% confidence intervals (CI) for schistosomiasis infection of individuals to demographic variables.**

Variable	No. of individuals	Infected n (%)	PR	95% CI	p-value
Sex					
Male	154	82(53.25)	1.43	1.18-1.73	0.0002
Female	446	166(37.22)	1.00		
Age group					
< 30	129	62(48.06)	1.44	1.07-1.95	0.0170
30 - 60	339	142(41.89)	1.26	0.96-1.65	0.0996
> 60	132	44(33.33)	1.00		
Sub-county of residence					
Rachuonyo North	300	145(48.33)	1.51	1.11-2.06	0.0088
Rarieda	200	71(35.50)	1.11	0.79-1.56	0.5512
Bondo	100	32(32.00)	1.00		

and sanitation variables considered, occupation, source of water for domestic use ~~and~~ the size of the households were significantly associated with prevalence of schistosomiasis. However, there was no significant association between the level of education, wealth class, and presence of latrine in the respective household and the prevalence of schistosomiasis. The infection prevalence among those individuals ~~who practiced fishing as the main source of income was~~ 1.54 (95% CI = 1.24 − 1.92) times the prevalence among those ~~who practiced other~~ occupations (apart from fishing and farming) . However, there was no difference in infection prevalence between those who practice farming and those who practiced other occupations as the main source of earning a living. Schistosomiasis prevalence among individuals who used unsafe water sources as the main source of water for domestic use was 1.28 (95% CI = 1.04 − 1.58) times the prevalence for those who used safe water sources as the main source of water for domestic use. With regard to housing conditions, infection prevalence for individuals who came from households with atleast 4 persons was 1.36 (95% CI = 1.08 − 1.71) times the prevalence for those who came from households with less than 4 persons. However, education level, socio-economic position and presence of latrine in the household where the individual came from were not significantly associated with infection prevalence (Table 2)

Table 2: **Prevalence ratios (PR) and 95% confidence intervals (CI) for schistosomiasis infection of individuals according to socioeconomic and sanitation characteristics.**

Variable	No. of individuals	Infected n (%)	PR	95% CI	p-value
Education level					
None	282	113(40.07)	0.94	0.78 - 1.14	0.5549
Atleast Primary	318	135(42.45)	1.00		
Occupation					
Fishing	94	57(60.64)	1.54	1.24 - 1.92	0.0001
Agriculture	229	82(35.81)	0.91	0.73 - 1.14	0.4150
All other occupations	277	109(39.35)	1.00		
Socio-economic status					
Poor	360	142(39.44)	0.89	0.74 - 1.08	0.2468
Less Poor	240	106(44.17)	1.00		
Water supply					
Unsafe	375	169(45.07)	1.28	1.04 - 1.58	0.0197
Safe	225	79(35.11)	1.00		
Household size					
Atleast 4 persons	416	187(44.95)	1.36	1.08 - 1.71	0.0098
$<$ 4 persons	184	61(33.15)	1.00		
Latrine present?					
No	136	55(40.44)	0.97	0.77 - 1.22	0.8111
Yes	464	193(41.59)	1.00		

Table 3 shows the results of the multivariable log binomial model for schistosomiasis infection. Several plausible interactions such as socioeconomic status and presence of latrine, socioeconomic status and highest education level, socioeconomic status and household size or socioeconomic status and the main source of water were included in the model containing all the independent variables. Among all the possible models, the model with

the lowest value with respect to SBC was selected but none of the interactions was present in the final model. Then, confounding was assessed by adding each of the variables considered in the univariate analysis but not included in the final multivariable analysis to the selected model. The variables gender, sub-county, number of persons per household, remained significantly associated with infection when controlling for the other variables in the model. Age was only significant in the model where education level and occupation were not included, thus we concluded that the association between age and schistosomiasis infection was confounded by occupation and education level. However, socioeconomic status remained non-significant even after including education level, occupation or presence/absence of latrine in the final model. Holding constant the other

Table 3: **Multivariate log binomial model of the association between demographic, socioeconomic and sanitation variables and schistosomiasis prevalence.**

**Variable**	**No. of individuals**	**Adj. PR**	**95% CI**	**p-value**
Sex				
Males	154	1.37	1.14 - 1.66	0.0010
Females	446	1.00		
Sub-county of residence				
Rachuonyo North	300	1.48	1.09 – 2.01	0.0131
Rarieda	200	1.23	0.87 - 1.73	0.2430
Bondo	100	1.00		
Socio-economic status				
Poor	360	0.92	0.77 – 1.11	0.3770
Less Poor	240	1.00		
Water supply				
Unsafe	375	1.19	0.96 - 1.47	0.1171
Safe	225	1.00		
Household size				
Atleast 4 persons	416	1.29	1.03 - 1.61	0.0300
< 4 persons	184	1.00		
Age group				
< 30	129	1.36	1.01-1.82	0.0419
30 - 60	339	1.22	0.93-1.60	0.1549
> 60	132	1.00		

predictors entered into the model, gender was strongly associated with *S. mansoni* infection. Males were 1.37 (95% CI = 1.14–1.66) times more likely to be infected when compared with females. The other variables or variable levels showing a significant or marginally significant association with infection when compared with the referent level were sub-county of residence, age-group and number of persons per household. Residents of Rachuonyo North sub-county were 1.48 (95% CI = 1.09 – 2.01) times more likely to be infected when compared to those from Bondo sub-county; individuals aged below 30 years were 1.36 (95% CI = 1.01 -1.82) times more likely to be infected as compared to those in the above 60 years while households with at least 4 persons were 1.29 (95% CI = 1.03 – 1.61) times more likely to be infected as compared to those with < 4 persons. The composite wealth (socioeconomic status) index generated using MCA was neither significant in the univariate analysis (Table 2) nor in the multivariate analysis (Table 3). Since all variables in the multivariable model were

considered potential risk factors for *S. mansoni* infection, egg counts (log transformed) were compared across each category of those variables. A linear mixed model with Tukey adjustment was used to compare geometric mean egg counts and the 95% confidence interval (CI) for each level of the selected variables. Geometric mean egg counts were significantly associated with gender (F-statistic=9.08, $p < 0.0029$) and marginally significant with sub-county of residence (F-statistic=14.19, $p < 0.001$) and age (F-statistic=4.19, p=0.0163). The Tukey multiple comparison procedure indicated that geometric mean egg counts among Rachuonyo North residents was significantly higher as compared for that among Rarieda sub-county residents. In addition, the mean EPG among Rarieda residents was also significantly low when compared to those among Bondo sub-county residents although the EPGs were not significantly different when Rachuonyo North was compared with Bondo. In comparing geometric EPGs among below 30 years, 30-60 years and above 60 age-groups, the Tukey multiple procedure indicated that the EPGs among those aged below 30 was significantly higher as compared to that for those aged between 30-60 years and that for those aged above 60 years. However, there was no significant difference in the EPGs among those aged between 30-60 when compared to those aged > 60 years. The overall statistics did not indicate any significant differences in the geometric mean egg counts with type of water supply (safe/unsafe), socio-economic status, or number of persons in a household (Table4)

Table 4: **Intensity of schistosomiasis infection of individuals expressed as geometric mean of *S. mansoni* eggs per gram of feces, by predictors in the multivariable model.**

Variable	No. Infected	Mean EPG (log_{10})	Geometric Mean EPG	95% CI	Turkey grouping*
Sex					
Males	82	4.24	80.55	57.31 - 113.23	A
Females	166	3.65	47.28	37.81 - 59.11	B
Overall F-statistic = 9.08; p = 0.0029					
Sub-county of residence					
Rachuonyo North	145	4.46	82.27	63.83 - 106.03	A
Rarieda	71	3.65	24.64	18.32 - 33.15	B
Bondo	32	4.02	59.47	40.58 - 87.16	A
Overall F-statistic = 14.19; p = < 0.001					
Socio-economic status					
Poor	142	3.89	53.47	41.39 - 69.06	A
Less Poor	106	4.02	60.18	45.45 - 79.69	A
Overall F-statistic = 0.44; p = 0.5066					
Water supply					
Unsafe	169	3.99	58.56	46.65 - 73.53	A
Safe	79	3.90	51.51	36.59 - 72.52	A
Overall F-statistic = 0.20; p = 0.6546					
Household size					
Atleast 4 persons	187	3.96	55.36	44.66 - 68.64	A
< 4 persons	61	3.94	58.98	39.50 - 88.06	A
Overall F-statistic = 0.01; p = 0.9132					
Age group					
< 30	62	4.37	77.82	53.29 - 113.63	B
30 - 60	142	3.92	56.38	44.04 - 72.18	A
> 60	44	3.55	34.36	21.91 - 53.89	A
Overall F-statistic = 4.19; p = 0.0163					

∗ *The mean EPG for various categories of each variable were tested for significant differences. Categories indicating no statistically significant difference within a variable display the same letter while those that are significantly different do not have the same letter.*

Discussion

This cross-sectional study conducted in Rachuonyo North, Rarieda and Bondo sub-counties, in rural settings proximate to Lake Victoria which had been associated with high endemicity of schistosomiasis in Western Kenya according to previous studies [27], [28], [29] evaluated the relationship between burden of *S. mansoni* infection and household SES index. This is the first published paper to assess the relationship between *S. mansoni* infection and SES using the MCA model to generate household wealth quintiles based on continuous and categorical variables with regard to household assets. Our findings indicate no difference in prevalence of *S.mansoni* between individuals in the poorest households compared to those from less-poor households. Among the socioeconomic indicators considered (wealth class, occupation, education level and number of persons per household), only number of persons per household and occupation turned out to be a major predictors of schistosomiasis occurrence in both the univariate and multivariate models. Prevalence of *S. mansoni* infection was significantly higher in Rachuonyo North sub-county compared to Rarieda and Bondo sub-counties. This could be due to higher number of intervention studies done in Bondo and Rarieda in the recent past hence leading to a remarkable decline in prevalence. These differences could be attributed to the focal distribution of *S. mansoni* and closer proximity of villages in Rachuonyo North compared to Rarieda and Bondo sub-counties to Lake Victoria. The association between number of persons per household and *s.mansoni* infection corroborates with the findings by Gazzinelli and colleagues stating that as the number of persons per room increased, probability of schistosomiasis occurrence increased, although this association was only marginal [30]. Findings from this analysis, however, contrast with those of studies by Sady and Dawaki which found no association between family size and *s.mansoni* infection [31], [32]. The Sady study, however, was limited to children aged $\leq$ 15 years while the Dawaki study was based on both children and adults (aged 1 - 90 years) whereas in the study by Gazzinelli the analysis was not restricted to a specific age group (based on both children and adults (aged 2 - 95 years)). In addition, both Sady and Dawaki studies were based on populations in which *S.mansoni* was relatively low, that is, 9.3% and 17.8%, respectively although they both used odds ratios from logistic regression to estimate the prevalence ratios unlike both the current study and Gazzinelli study used Prevalence ratios from a log binomial regression model. With regard to the association between schistosomiasis prevalence and wealth classes we found no significant association in prevalence between socioeconomic groups. These findings are consistent with the results from a study conducted in Tanzania that used PCA to calculate the quintiles [8]. However, according to recent literature PCA models are weaker compared to MCA due to their inability to accommodate continuous variables such as number of assets owned and could possibly lead to generation of low asset weights, hence difficulty in determining clear wealth quintile cut-offs particularly in settings where most households have the same assets and therefore the same or very similar SES scores [20], [16]. This study therefore applied MCA for assessment of socioeconomic status in relation to schistosomiasis prevalence inequalities.Our results however contradict the results from a study by Muhumuza and a systematic review by Houweling ([6], [34] both of which found out that wealth index is an independent predictor of schistosomiasis occurrence. Muhumuza's study

generated wealth classes by assigning arbitrary scores to household assets, for example, ~~for materials used for walls of main house~~ (no bricks = 1, unburnt bricks = 2, burnt bricks with mud = 3, burnt bricks,stones with cement = 4), this could however at times generate subjective classes when applied to other settings. Houweling's systematic review only included international publications in the last decade from the time of publication and studies conducted in the 20 countries with the highest burden for each NTD and could possibly have suffered from publication and review bias. Other studies that found a significant association between socioeconomic status and schistosomiasis prevalence include those by Sady and Dawaki ~~which however~~ relied on individual income levels to create socioeconomic status indices. This method of generating socioeconomic indices could at times suffer from recall bias, seasonality and variation as a result of timing for data collection activity [31, 32]. Our findings may be attributed to the poor socioeconomic environment in villages proximate to Lake Victoria. The absence of socioeconomic effect could be attributed to the widespread poverty and the rural way of life which tend to put the whole community at risk of infection. The current study found a significant association between occupation and schistosomiasis whereby those who practice fishing were at greater risk of being infected with schistosomiasis compared to those who practice farming among other occupational activities. This could be due to possible contact with infested water in the course of duty. Previous studies by Salawu, Martin and Gazzinelli have also pointed out occupation to be a significant predictor of Schistosomiasis [35], [36], [30]. Salawu's study~~, however,~~ was only based on pregnant women aged ≥ 15; Martin's study did not involve any specific age bracket (population aged between 0 - 96) while Gazzinelli's study took into consideration possibility of multiple sampling of individuals within a households by analyzing correlation structures between samples obtained from the same household. No significant differences in *S.Mansoni* prevalence between individuals who possessed atleast primary education and those who were less educated was observed in this rural western Kenya community. These findings are in agreement with those from a study conducted in North Central Nigeria that involved both children and adults [32], and a cross-sectional community-based field study conducted ~~between February 1, 2010, and February 15, 2011~~ among pregnant women in Nigeria [35]. These findings however contradict the findings of: a cross-sectional community-based study carried out among children aged ≤ 15 years in rural areas in Yemen [31], a systematic literature review on the socioeconomic distribution within endemic countries of LF, onchocerciasis, schistosomiasis, STH, trachoma, Chagas' disease, HAT, leprosy, and VL which ~~however~~ only involved publications between 2004–2013 in countries comprising almost 90% of the global burden for the studied NTDs [34] and a study conducted in a low range of medium prevalence for schistosomiasis in South East Brazil which also involved both children and adults [36]. Among the demographic variables considered, our study identified gender to be the strongest predictor of *S.mansoni* infection. This association has also been identified by other studies in Nigeria and Brazil. [32, 36].

Conclusion

Our primary findings could have several implications for schistosomiasis control in rural areas of Western Kenya. First, since *S. mansoni* infec-

tion was not significantly associated with socioeconomic status as a result of widespread poverty indicates that only widely based rural development initiatives aimed at reducing population dependency on unsafe water sources for domestic, recreational and occupational purposes could benefit the entire population. These initiatives could include basic public health interventions such as provision of an adequate piped water supply, sanitation facilities and health education may thus benefit schistosomiasis control programs in this rural setting. Secondly, while number of persons per household could be a useful indicator of schistosomiasis risk in a subsistence economy, this association primarily indicates a predominantly demographic or density rather than a socioeconomic influence. Thirdly, it is worth noting that with the rolling out of Phase I and Phase II rural electrification programmes in many settings, the presence/absence of electricity in a household does not necessarily indicate socioeconomic status since with the programme, electricity is made available notwithstanding one's economic strength. Lastly, this study demonstrates that the MCA model can be a useful tool for assessing Schistosomiasis-related health disparities at the household level and for monitoring progress towards achieving equitable access to health services and distribution of control and elimination interventions with regard to national and global health and development goals.

Limitations

We acknowledge some limitations of our methodology. This study had to rely on a single faecal sample collection and a single Kato-Katz smear instead of the ideal three consecutive samples and multiple Kato-Katz smears examination [38]. Therefore, the prevalence and intensity of schistosomiasis are likely to be underestimated due to the temporal variation in egg excretion over hours and days. Moreover, our findings were based on a single cross-sectional survey hence the inability to evaluate cause-and-effect of SES on Schistosmiasis prevalence over time. A longitudinal or trend analysis of repeated surveys would be ideal in analysing changes in SES and monitoring the gap in schistosmiasis prevalence between the poorest and less-poor households.

Funding

Financial support to conduct this study was received from University of Georgia Research Foundation, Inc. which was funded by the Bill & Melinda Gates Foundation.

Acknowledgements

We ~~hereby~~ express our gratitude to all interviewees for their participation in this study. We are also grateful for the continuous endeavours of fieldworkers and laboratory technicians of the Kenya Medical Research Institute (KEMRI) NTD branch without whose efforts this research would not have been ~~termed~~ a success, and to the members of the Health Department of Siaya and Homabay Counties for their enduring collaboration. We gratefully acknowledge the financial support received from the Bill & Melinda Gates Foundation.

References

[1] Mondiale de la Santé, O., & World Health Organization. *Schistosomiasis and soil-transmitted helminthiases: number of people treated in 2016–Schistosomiase et géohelminthiases: nombre de personnes traitées en 2016.* Weekly Epidemiological Record= Relevé épidémiologique hebdomadaire, 92(49), 749-760, 2017

[2] Abajobir AA, Abate KH, Abbafati C, Abbas KM, Abd-Allah F, Abdulkader RS, et al. *Global, regional, and national disability-adjusted life-years (DALYs) for 333 diseases and injuries and healthy life expectancy (HALE) for 195 countries and territories, 1990–2016: a systematic analysis for the Global Burden of Disease Study 2016.* Lancet. 2017;390: 1260–1344. pmid:28919118

[3] Hotez, P. J., & Kamath, A. (2009) *Neglected tropical diseases in sub-Saharan Africa: review of their prevalence, distribution, and disease burden.* PLoS neglected tropical diseases, 3(8), e412

[4] Daumerie, D., & Savioli, L. (2010) *Working to overcome the global impact of neglected tropical diseases: first WHO report on neglected tropical diseases (Vol. 1)* World Health Organization.

[5] Schistosomiasis,W.H.O.(2018) *FactSheet No 115.World Health Organization.*Available at http://www.who.int/mediacentre/factsheets/fs115/en.

[6] Muhumuza, S., Kitimbo, G., Oryema-Lalobo, M., & Nuwaha, F. (2009). *Association between socio economic status and schistosomiasis infection in Jinja District, Uganda.* Tropical Medicine & International Health, 14(6), 612-619.

[7] Mwanga, J. R., Kaatano, G. M., Siza, J. E., Chang, S. Y., Ko, Y., Kullaya, C. M., ... & Min, D. Y. (2015). *Improved Socio-Economic Status of a Community Population Following Schistosomiasis and Intestinal Worm Control Interventions on Kome Island, North-Western Tanzania.* The Korean journal of parasitology, 53(5), 553.

[8] Donohue, R. E., Mashoto, K. O., Mubyazi, G. M., Madon, S., Malecela, M. N., & Michael, E. (2017). *Biosocial Determinants of Persistent Schistosomiasis among Schoolchildren in Tanzania despite Repeated Treatment.* Tropical Medicine and Infectious Disease, 2(4), 61.

[9] Nagi, S., Chadeka, E. A., Sunahara, T., Mutungi, F., Justin, Y. K. D., Kaneko, S., ... & Shimada, M. (2014). *Risk factors and spatial distribution of Schistosoma mansoni infection among primary school children in Mbita District, Western Kenya.* PLoS neglected tropical diseases, 8(7), e2991.

[10] Filmer, D., & Scott, K. (2008). *Assessing asset indices.* The World Bank.

[11] Vyas, S., & Kumaranayake, L. (2006). *Constructing socio-economic status indices: how to use principal components analysis.* Health policy and planning, 21(6), 459-468.

[12] Filmer, D., & Pritchett, L. (1998). *Estimating Wealth Effects without Expenditure Data – or Tears: An Application to Educational Enrollments in States of India.* Policy Research Working Papers No. 1994.

[13] Filmer, D. & Pritchett, L. (2001). *Estimating wealth effect without expenditure data — or tears: An application to educational enrollments in states of India*, Demography 38, 115–132.

[14] Kolenikov, S., & Angeles, G. (2009). *Socioeconomic status measurement with discrete proxy variables: Is principal component analysis a reliable answer?* Review of Income and Wealth, 55(1), 128-165.

[15] Amek, N., Vounatsou, P., Obonyo, B., Hamel, M., Odhiambo, F., Slutsker, L., & Laserson, K. (2015). *Using health and demographic surveillance system (HDSS) data to analyze geographical distribution of socio-economic status; an experience from KEMRI/CDC HDSS.* Acta tropica, 144, 24-30.

[16] Su-Myat, K. K., de Tibeiro, J. J., & Kumar, P. (2012). *An integrated approach to regression analysis in multiple correspondence analysis and copula-based models.* Journal of Statistics Applications & Probability, 1(2), 1.

[17] Handzel, T., Karanja, D. M., Addiss, D. G., Hightower, A. W., Rosen, D. H., Colley, D. G., ... & Secor, W. E. (2003). *Geographic distribution of schistosomiasis and soil-transmitted helminths in Western Kenya: implications for anthelminthic mass treatment.* The American journal of tropical medicine and hygiene, 69(3), 318-323.

[18] Le Roux, B., & Rouanet, H. (2004). *Geometric data analysis: from correspondence analysis to structured data analysis.* Springer Science & Business Media.

[19] Wiegand, R. E. (2017). *A Persistent Hotspot of Schistosoma mansoni Infection in a Five-Year Randomized Trial of Praziquantel Preventative Chemotherapy Strategies.*

[20] Abdi, H., & Valentin, D. (2007). *Multiple correspondence analysis.* Encyclopedia of measurement and statistics, 651-657

[21] Montresor, A., Crompton, D. W., Hall, A., Bundy, D. A., Savioli, L., & World Health Organization(1998). *Guidelines for the evaluation of soil-transmitted helminthiasis and schistosomiasis at community level: a guide for managers of control programmes.*

[22] Skove, T., Deddens, J., Petersen, M. R., & Endahl, L. (1998). *Prevalence proportion ratios: estimation and hypothesis testing.* International journal of epidemiology, 27(1), 91-95.

[23] Coutinho, L., Scazufca, M., & Menezes, P. R. (2008). *Methods for estimating prevalence ratios in cross-sectional studies.* Revista de saude publica, 42(6), 992-998.

[24] Deddens, J. A., & Petersen, M. R. (2008). *Approaches for estimating prevalence ratios. Occupational and environmental medicine*, 65(7), 501-506.

[25] Beal, D. J. (2007). *Information criteria methods in SAS for multiple linear regression models.* 15th Annual SouthEast SAS Users Group (SESUG) Proceedings, South Carolina, Paper SA05.

[26] Westfall, P. H., Tobias, R. D., & Wolfinger, R. D. (2011). *Multiple comparisons and multiple tests using SAS.* SAS Institute.

[27] Odiere, M. R., Rawago, F. O., Ombok, M., Secor, W. E., Karanja, D. M., Mwinzi, P. N., ... & Won, K. (2012). *High prevalence of schistosomiasis in Mbita and its adjacent islands of Lake Victoria, western Kenya.* Parasites & vectors, 5(1), 278.

[28] Adoka, S. O., Anyona, D. N., Abuom, P. O., Dida, G. O., Karanja, D., Vulule, J. M., ... & Ofulla, A. V. O. (2014). *Community perceptions of schistosomiasis transmission, prevalence and control in relation to aquatic habitats in the Lake Victoria basin of Kenya.* East African medical journal, 91(7), 232-244.

[29] Amollo, D. A., Kihara, J. H., Kombe, Y., & Karanja, S. M. (2013). *Prevalence and intensity of single and mixed schistosoma mansoni and schistosoma haematobium infections in primary school children in Rachuonyo North District, Homabay County, Western Kenya.* East African medical journal, 90(2), 36-44

[30] Gazzinelli, A., Velasquez-Melendez, G., Crawford, S. B., LoVerde, P. T., Correa-Oliveira, R., & Kloos, H. (2006). *Socioeconomic determinants of schistosomiasis in a poor rural area in Brazil.* Acta Tropica, 99(2-3), 260-271.

[31] Sady, H., Al-Mekhlafi, H. M., Mahdy, M. A., Lim, Y. A., Mahmud, R., & Surin, J. (2013). *Prevalence and associated factors of schistosomiasis among children in Yemen: implications for an effective control programme.* PLoS neglected tropical diseases, 7(8), e2377.

[32] Dawaki, S., Al-Mekhlafi, H. M., Ithoi, I., Ibrahim, J., Abdulsalam, A. M., Ahmed, A., ... & Nasr, N. A. (2016). *Prevalence and risk factors of schistosomiasis among hausa communities in Kano state, Nigeria.* Revista do Instituto de Medicina Tropical de São Paulo, 58.

[33] Su-Myat, K. K., de Tibeiro, J. J., & Kumar, P. (2012). *An integrated approach to regression analysis in multiple correspondence analysis and copula based models.* Journal of Statistics Applications & Probability, 1(2), 1.

[34] Houweling, T. A., Karim-Kos, H. E., Kulik, M. C., Stolk, W. A., Haagsma, J. A., Lenk, E. J., ... & de Vlas, S. J. (2016). *Socioeconomic inequalities in neglected tropical diseases: a systematic review.* PLoS neglected tropical diseases, 10(5), e0004546.

[35] Salawu, O. T., & Odaibo, A. B. (2016). *Schistosomiasis transmission; socio-demographic, knowledge and practices as transmission risk factors in pregnant women.* Journal of parasitic diseases, 40(1), 93-99.

[36] Enk, M. J., Lima, A. C. L., Barros, H. D. S., Massara, C. L., Coelho, P. M. Z., & Schall, V. T. (2010). *Factors related to transmission of and infection with Schistosoma mansoni in a village in the South-eastern Region of Brazil.* Memórias do Instituto Oswaldo Cruz, 105(4), 570-577.

[37] Yimen, N., Hamandjoda, O., Meva'a, L., Ndzana, B., & Nganhou, J. (2018). *Analyzing of a Photovoltaic/Wind/Biogas/Pumped-Hydro Off-Grid Hybrid System for Rural Electrification in Sub-Saharan Africa—Case Study of Djoundé in Northern Cameroon.* Energies, 11(10), 2644.

[38] Berhe, N., Medhin, G., Erko, B., Smith, T., Gedamu, S., Bereded, D., ... & Gundersen, S. G. (2004). *Variations in helminth faecal egg counts in Kato–Katz thick smears and their implications in assessing infection status with Schistosoma mansoni.* Acta Tropica, 92(3), 205-212.
