# Supplementary material for: Socioeconomic determinants of Schistosoma mansoni infection using multiple correspondence analysis among rural western Kenyan communities: Evidence from a household-based study
Source: PLoS One. 2021 Jun 23;16(6):e0253041. doi: 10.1371/journal.pone.0253041 (PMC8221481; doi:10.1371/journal.pone.0253041)
Supplement: Attachment — Submitted filename: PONE-D-20-05468 R1 editors comments.docx [file pone.0253041.s005.docx]

**Socioeconomic determinants of Schistosoma mansoni infection using multiple correspondence analysis among rural western Kenyan communities: evidence a household-based study.**

Full Title: There are no line numbers in the Abstract making review and responses more challenging. The word 'from' is missing in this title.

Abstract

normally this is written as 'and/or'

Methodology

purposely selected for this study.

In the Results you describe convenience sampling. Which was it: random or convenience?

Did you take the average for intensity of infection form these 4 slides?

I believe you mean that an interview with a questionnaire based upon MCA inductors was conducted.

I think you mean age group and should define the ages used in these groups.

do you mean intensity (epg)?

Principal Findings

What ages?

What is the definition of a hotspot village? Be consistent throughout the manuscript if you are going to capitalise these words or not

Please use the WHO clarifications in brackets (unprotected well, lakes and river)

This sentence can begin with Intensity

Was 'also'

This last sentence would flow better before the sentence about intensity UNLESS you want to make the statement that both prevalence AND intensity were not significant.

Conclusion

Or the sample size was small? Where have you justified the sample size of 600 adults? If the sampling was by 'convenience' that would contribute to the lack of significant findings.

Also intensity was light which makes discerning differences more nuanced than this sample size would achieve if convenience sampling was used.

Introduction

L 10: Who regards it as a social disease?

L 24: The word 'different' is unnecessary

L 25: The words 'which is' are unnecessary

L 40: Better to re-phrase this as low-and middle-income countries (LMICs)

L 45: Again, you don't need to capitalise Cupboard or Radio

L 47: Why bring in 'developing' when you have already mentions LMICs

L 53: Rather than 'rich-poor' would 'wealth' be more appropriate?

L 73: You have already introduce the acronym MCA so please use it throughout the remaining manuscript.

L 79: Please be consistent with the references before or after the full stop: read intructions for authors.

L 80: Unless you are going to use this acronym another 3 times please do not introduce it.

Materials and Methods

L 109: I suggest combining the data and saying 'ranging from xxx to xxx' rather than presenting them separately as they have no further part to play in the analysis and findings.

L 115: Any reader with a background in NTDs will be wanting to understand why you are only considering S. mansoni and not S. haematobium as well. Explain why urine was not examined for S. haematobium and other sources of data supporting that such as mapping oand/or impact assessments.

Study Design

L 125: please delete the space between the numerical symbols and the number on all these.

L 130: The abstract reported 'random' sampling. Which was it?

L 132: Were the household selected by convenience or randomly? Once selected were the adults within the household selected by convenience or random. If by random explain the process.

L 137: Since you are not reporting on S. haem why did you include this. Did you find any S. mansoni in the urine samples? If you relied on CCA how did you judge or adjust intensity of infection?

Parasitological Assessment

L 142: If they had to produce a stool sample on the same day as the study there is a risk that it was not actually being their sample. Normally containers are provided the night before the mitigate this risk!

L 146: This should not have been appropriately. You cannot assess epg by 'approximately'

L 150: Did you take an average of these counts as your final figure? If not how was EPG derived?

L 159: How have you reconciled intensity if some individuals were only confirmed positive by CCA? Please clearly identify in a footnote to the table how many positive were from CCA ONLY.

Collection of Socioeconomic Data

L 164: sex (not gender)

L 168 : Wouldn’t' wealth index be more appropriate?

Statistical Analysis

L 212: Wouldn’t' wealth index be more appropriate?

L 246: Introducing all these models and discussion of their merits is inappropriate in methods. transfer this to either background or discussion. In methods just describe the method (MCA) used in this study.

Ethical Considerations

L 278: You need to move here the orientation of participants and how consent was obtained, recorded, anonymized and stored.

Result

L 288: Pease define this a light intensity infection for S. mansoni

L 293: So using the WHO calcifications of intensity what proportion of those infected have moderate/heavy intensity infections?

Association between infection and variable

L 298: Pease remove the space before and after the '-' for all these CI intervals in ALL the following results. You have presented CIs neatly in Table 1 without these unnecessary spaces.

L 304: Many minor typos like this must get corrected before the next review. Please use spelling and grammar check before re-submission.

L 321: Missing space: at least. Numbers less than ten should be written e.g. four

L 333: sex

L 347 Table 3: Inconsistent use of spacers before and after ‘-‘

L 350: This is reverting to methods rather than focusing on the results. The whole description and discussion of MCA versus other methods is too long and spills over from introduction to methods and now even into results.

L 371: capitalization

L 374: space

L 374 Table 4: Inconsistent use of spaces before and after '-'

Discussion

L 370: Refer to instructions for authors regarding how multiple references should be formatted [30, 31, 32].

L 393: How would the number of studies impact prevalence?

L 398: Capital 'S'

L 404: Household size rather than family size.

L 405: formatting []

L 407: Redundant spaces

L 413: lower case 'p'

L 425: This sentence is repetitious to arguments already explained.

L 429: [6, 37]

L 432: Unnecessary spaces before and after

L 451: formatting

L 452: space

L 453: spaces

L 457: Lover case 'm'

L 474: Sex

Limitations

L 479: You described 4 slides not one

L 482: Why only underestimated?

L 483: The abstract clearly states random sampling

Also unclear how many cases were diagnosed on urine CCA and how that was used to assess intensity.

Conclusion

L491: Too long. You conclusion should be based on whether or not MCA was a useful predictor of S mansoni prevalence or intensity. If you had used robust random sampling it might have been.
