# Supplementary material for: Socioeconomic determinants of Schistosoma mansoni infection using multiple correspondence analysis among rural western Kenyan communities: Evidence from a household-based study
Source: PLoS One. 2021 Jun 23;16(6):e0253041. doi: 10.1371/journal.pone.0253041 (PMC8221481; doi:10.1371/journal.pone.0253041)
Supplement: Attachment — Submitted filename: reviewer_comments.docx [file pone.0253041.s006.docx]

To:

The Editor, *PLOS ONE*

Dear Sir/Madam,

Thank you for the comments for our manuscript titled “***Socioeconomic Determinants of Schistosoma mansoni infection using multiple correspondence analysis among rural western Kenyan communities: evidence a household-based study.***”. The comments from the reviewers are much appreciated. The manuscript has been revised taking into account the suggested adjustments.

Full Title: There are no line numbers in the Abstract making review and responses more challenging. The word 'from' is missing in this title.

***Line numbers have been introduced from the abstract section throughout the entire document***

Abstract

normally this is written as 'and/or'

***Phrase has been reworded in line 11***

Methodology

purposely selected for this study.

In the Results you describe convenience sampling. Which was it: random or convenience?

***Rewording has been done to emphasize that random sampling was done to identify the villages while convenience sampling was used to identify the 50 participants in each village in Lines 13-16***

Did you take the average for intensity of infection form these 4 slides? ***Yes. Line 22-23***

I believe you mean that an interview with a questionnaire based upon MCA inductors was conducted***. Yes. Editting has been done in lines 20,21***

I think you mean age group and should define the ages used in these groups. ***Editting has been done in lines 25***

do you mean intensity (epg)? ***Yes, Lines 27,28***

Principal Findings

What ages?

What is the definition of a hotspot village? Be consistent throughout the manuscript if you are going to capitalise these words or not ***Throughout the document, the first letter has been capitalized for Hotspots and Respondents***

Please use the WHO clarifications in brackets (unprotected well, lakes and river***) Editting done in line 33***

This sentence can begin with Intensity ***Editting done in line 41***

Was 'also' ***‘Also’ has been inserted in line 42***

This last sentence would flow better before the sentence about intensity UNLESS you want to make the statement that both prevalence AND intensity were not significant. ***Editting has been done accordingly***

Conclusion

Or the sample size was small? Where have you justified the sample size of 600 adults? If the sampling was by 'convenience' that would contribute to the lack of significant findings.

Also intensity was light which makes discerning differences more nuanced than this sample size would achieve if convenience sampling was used. ***The inadequacy of convenience sampling has been included as a possible cause for non-significant results in line 47***

Introduction

L 10: Who regards it as a social disease? ***In line 62, citation has been added for one of the studies that proved that schistosomiasis is a social disease***

L 24: The word 'different' is unnecessary ***The word has been dropped in line 76***

L 25: The words 'which is' are unnecessary ***The phrase*** 'which is' ***has been dropped in line 77***

L 40: Better to re-phrase this as low-and middle-income countries (LMICs) ***Editting has been done in line 91***

L 45: Again, you don't need to capitalise Cupboard or Radio ***Editting has been done in line 96***

L 47: Why bring in 'developing' when you have already mentions LMICs ***Editting has been done in line 98***

L 53: Rather than 'rich-poor' would 'wealth' be more appropriate? ***'rich-poor' has been replaced with 'wealth' in line 104***

L 73: You have already introduce the acronym MCA so please use it throughout the remaining manuscript. ***MCA has been introduced in line 6 and an acronym used elsewhere in the manuscript***

L 79: Please be consistent with the references before or after the full stop: read intructions for authors. ***Editting has been done to ensure all references appear before full stop***

L 80: Unless you are going to use this acronym another 3 times please do not introduce it. ***The acronym ‘CA’ has been dropped in line 130***

Materials and Methods

L 109: I suggest combining the data and saying 'ranging from xxx to xxx' rather than presenting them separately as they have no further part to play in the analysis and findings. ***The statements have been combined in lines 168-169***

L 115: Any reader with a background in NTDs will be wanting to understand why you are only considering S. mansoni and not S. haematobium as well. Explain why urine was not examined for S. haematobium and other sources of data supporting that such as mapping oand/or impact assessments. ***A statement has been added in lines 223 and 224 that STH and urine results were not reported in this paper. The study’s main focus was s. mansoni. The urine was used for M-reader and POC-CCA which also tests for s. mansoni (beyond the scope of this paper). The focus of this study was s.mansoni results form Kato katz technique.***

Study Design

L 125: please delete the space between the numerical symbols and the number on all these. ***All unnecessary spaces removed***

L 130: The abstract reported 'random' sampling. Which was it? ***In lines 188 and 190, clarification has been made that random sampling was used to identify the 12 villages while convenience sampling was used to identify individual participants, the same concept has been reflected in the abstract***

L 132: Were the household selected by convenience or randomly? Once selected were the adults within the household selected by convenience or random. If by random explain the process. ***The households were identified by convenience sampling. Only one adult was interviewed per household, line 191***

L 137: Since you are not reporting on S. haem why did you include this. Did you find any S. mansoni in the urine samples? If you relied on CCA how did you judge or adjust intensity of infection? ***For intensity of infection, we relied on KK results***

Parasitological Assessment

L 142: If they had to produce a stool sample on the same day as the study there is a risk that it was not actually being their sample. Normally containers are provided the night before the mitigate this risk! ***In line 202, clarification has been provided that the plastic containers were provided a day prior to survey date***

L 146: This should not have been appropriately. You cannot assess epg by 'approximately' ***The word approximately has been dropped, line 208***

L 150: Did you take an average of these counts as your final figure? If not, how was EPG derived? ***Yes, clarification provided in line 212***

L 159: How have you reconciled intensity if some individuals were only confirmed positive by CCA? Please clearly identify in a footnote to the table how many positive were from CCA ONLY. ***Clarification has been made in line 217 that all statistical analysis in this paper were based on stool KK results***

Collection of Socioeconomic Data

L 164: sex (not gender) ***editing done, line 229***

L 168: Wouldn’t' wealth index be more appropriate? ***editing done, line 233***

Statistical Analysis

L 212: Wouldn’t' wealth index be more appropriate? ***editing done, line 277***

L 246: Introducing all these models and discussion of their merits is inappropriate in methods. transfer this to either background or discussion. ***In methods just describe the method (MCA) used in this study. Unnecessary details have been dropped***

Ethical Considerations

L 278: You need to move here the orientation of participants and how consent was obtained, recorded, anonymized and stored. ***Details have been provided in lines 337-339***

Result

L 288: Pease define this a light intensity infection for S. mansoni

L 293: So using the WHO calcifications of intensity what proportion of those infected have moderate/heavy intensity infections? ***Proportions provided in lines 347***

Association between infection and variable

L 298: Pease remove the space before and after the '-' for all these CI intervals in ALL the following results. You have presented CIs neatly in Table 1 without these unnecessary spaces. ***Unnecessary spaces removed***

L 304: Many minor typos like this must get corrected before the next review. Please use spelling and grammar check before re-submission. ***Errors resolved line 365***

L 321: Missing space: at least. Numbers less than ten should be written e.g., four ***Space added, 4 replaced with ‘four’***

L 333: sex ***gender replaced with sex, line 395***

L 347 Table 3: Inconsistent use of spacers before and after ‘-‘ ***Unnecessary spaces removed, table 3***

L 350: This is reverting to methods rather than focusing on the results. The whole description and discussion of MCA versus other methods is too long and spills over from introduction to methods and now even into results. ***Section moved to methods to reduce redundancy***

L 371: capitalization Hotpot and Responder have been used in the proper form throughout the document

L 374: space ***Space introduced between ‘table’ and ‘4’***

L 374 Table 4: Inconsistent use of spaces before and after '-' ***Unnecessary spaces removed, table 4***

Discussion

L 370: Refer to instructions for authors regarding how multiple references should be formatted [30, 31, 32]. ***Editing done, line 435***

L 393: How would the number of studies impact prevalence? ***Editing has been done to reflect intervention programs***

L 398: Capital 'S' ***‘S’ capitalized, line 451***

L 404: Household size rather than family size. ***‘Family size’ replaced with ‘household size’, line 457***

L 405: formatting [] ***Editing done, line 458***

L 407: Redundant spaces ***spaces removed in ranges***

L 413: lower case 'p' ***editing done, line 466***

L 425: This sentence is repetitious to arguments already explained***. Repetition removed***

L 429: [6, 37] ***Editing done, line 475***

L 432: Unnecessary spaces before and after ***Unnecessary spaces removed, line 478***

L 451: formatting ***Editing done, line 498***

L 452: space ***Editing done, line 499***

L 453: spaces ***Editing done, line 500***

L 457: Lover case 'm' ***Editing done, line 504***

L 474: Sex ***Editing done, line 520***

Limitations

L 479: You described 4 slides not one ***Editing done to reflect 1 stool sample rather than 3 ideal samples line 526***

L 482: Why only underestimated***? ‘Underestimated/overestimated’ to replace ‘underestimated’***

L 483: The abstract clearly states random sampling

Also unclear how many cases were diagnosed on urine CCA and how that was used to assess intensity. ***Clarification has been provided so the abstract, methods and limitations tell the same story.***

Conclusion

L491: Too long. You conclusion should be based on whether or not MCA was a useful predictor of S mansoni prevalence or intensity. If you had used robust random sampling it might have been. ***Section shortened for clarity***

We hope our revisions have adequately addressed the issues raised by reviewers and look forward to hearing from you.

Isaiah Omondi

Corresponding author
