# Supplementary material for: Socioeconomic determinants of Schistosoma mansoni infection using multiple correspondence analysis among rural western Kenyan communities: Evidence from a household-based study
Source: PLoS One. 2021 Jun 23;16(6):e0253041. doi: 10.1371/journal.pone.0253041 (PMC8221481; doi:10.1371/journal.pone.0253041)
Supplement: Attachment — Submitted filename: reviewer_comments.docx [file pone.0253041.s007.docx]

To:

The Editor, *PLOS ONE*

Dear Sir/Madam,

Thank you for the comments for our manuscript titled “***Socioeconomic Determinants of Schistosoma mansoni infection using multiple correspondence analysis among rural western Kenyan communities: evidence a household-based study.***”. The comments from the reviewers are much appreciated. The manuscript has been revised taking into account the suggested adjustments.

Line 20: ‘indicators’ has replaced ‘inductors’

Line 161: ‘Schistosomiasis’ has been replaced with ‘schistosomiasis’

Line 136: sentence rephrased to read ‘Yet, such data if properly analysed would be necessary for monitoring health inequalities and the impact of schistosomiasis control interventions at microeconomic level’

Line 303-304: ‘SES’ has replaces ‘socioeconomic status’

Lines 357 & 358: Sentence rephrased to read “the prevalence among males was 1.43(95% CI=1.18-1.73) times higher compared to the prevalence among females”
Line 389: ‘SES’ has replaces ‘socioeconomic status’
Lines 419-421: Sentence rephrased to read “…..the EPGs among those aged below 30 was

significantly higher as compared to those aged between 30-60 years and above 60 years”.

Lines 423-425: Sentence rephrased to read “The EPGs among the residents of Hotspot villages was significantly higher compared to residents of Responder villages”.
Line 427: ‘SES’ has replaces ‘socioeconomic status’


Discussion
Lines 505-509: Statement rephrased to read: “Our findings also contrast the findings of a systematic literature review on the socioeconomic distribution within endemic countries of lymphatic filariasis(LF), onchocerciasis, schistosomiasis, STH, trachoma, Chagas' disease, Human African Tripanosomiasis(HAT), leprosy, and visceral leishmaniasis(VL) which involved publications between 2004-2013 in countries comprising almost 90% of the global burden for the studied Neglected Tropical Diseases(NTDs)” .

We hope our revisions have adequately addressed the issues raised by reviewers and look forward to hearing from you.

Isaiah Omondi

Corresponding author
