# Supplementary material for: Socioeconomic determinants of Schistosoma mansoni infection using multiple correspondence analysis among rural western Kenyan communities: Evidence from a household-based study
Source: PLoS One. 2021 Jun 23;16(6):e0253041. doi: 10.1371/journal.pone.0253041 (PMC8221481; doi:10.1371/journal.pone.0253041)
Supplement: Attachment — Submitted filename: reviewer comments(1).docx [file pone.0253041.s004.docx]

To:

The Editor, *PLOS ONE*

Dear Sir/Madam,

Thank you for the comments for our manuscript titled “***Socioeconomic Determinants of Schistosoma mansoni infection using multiple correspondence analysis among rural western Kenyan communities: evidence a household-based study.***”. The comments from the reviewers are much appreciated. The manuscript has been revised taking into account the suggested adjustments.

**The reviewer preferred to see the use of S.mansoni addressed thoughout the manuscript**.

- Clarification have been made on all instances where schistosomiasis had been used instead of s. mansoni. S.mansoni has then been used to replace schistosomiasis. In addition, the full-term Schistosoma mansoni has been used in the title and the first time in the Abstract and in the main text after that it has been abbreviated to S. mansoni. The ‘S’ has been capitalized; the ‘M’ not capitalized. These changes have been effected on the following lines Abstract Line 6, All table titles1-4. L76, 84,85,86, 97,102, 106, 116, 287, 305, 418, 438, 476, 478, 449

**The reviewer questioned the clarity of sample size of 50 per stratum and then 600 stool samples represented.**

- Clarification has been provided to indicate that from each of the strata (Hotspot and Responder), 6 villages were selected and then from each village, 50 participants were selected. Hence the sample size 600 (2x6x50).

**The reviewer indicated that although urine samples had been collected, haematobium results were not referenced**

- Clarification has been provided to indicate that the urine sample was used to evaluate some techniques of diagnosing S. mansoni such as POC-CCA and M-Reader but not urine filtration. Therefore S. haematobium was not evaluated in this particular study. The above-named techniques were part of the main study from which this paper is developed.

**The reviewer questioned why there were no comparisons between hotspot and responders.**

- Comparisons have been added on the result section

**The reviewer questioned the clarity of the title.**

The title has been revised to “Socioeconomic determinants of Schistosoma mansoni infection using multiple correspondence analysis among rural western Kenyan communities: evidence from a household-based study.”

**Abstract, Background: The reviewer suggested space be created between “(MCA)” and “can” and also the original hypothesis stated**

Space has been introduced in line 5 as per the suggestion. The original hypothesis too has been stated in this section that MCA would be a useful predictor of S. mansoni prevalence and (or) intensity.

**Abstract, Methods: The reviewer needed clarification on the phrase ‘over the years’ in line 3, clarification on how the sample size of 600 was obtained and what the urine sample was used for if not to evaluate s. haematobium.**

The term over the years has been reworded to reflect the period from 2011 to 2015.

Clarification has been provided to indicate that from each of the strata (Hotspot and Responder), 6 villages were selected and then from each village, 50 participants were selected. Hence the sample size 600 (2x6x50)

Clarification has been provided to indicate that the urine sample was used to evaluate some techniques of diagnosing S. mansoni such as POC-CCA and M-Reader but not urine filtration

**Abstract, Findings: The reviewer recommended the presentation of comparisons between Hotspot and Responder villages and also presentation of results from univariate analyses**

Statistical analysis was revised to accommodate the comparison between Hotspots and Responder villages, the results have been reported as per the univariate and multivariate model. Significant differences in S.mansoni infection intensities have also been reported. In addition, when reporting difference in prevalence across wealth quintiles, a line has been introduced to explain that the wealth quintiles were broadly divided into ‘poor’ and ‘less poor’ categories.

**Abstract, Conclusion: The reviewer recommended this section to be presented in relation to the original hypothesis**

This section has been reworded to relate it to the study hypothesis

**Methods: The reviewer recommended that lines 173-181 would better suit the Background section**

The lines in question have been moved to the Background section

**Minor Comments**

In line 120 of the original manuscript, ‘other’ has been replaced with ‘another’

In line 121 of the original manuscript, clarification has been provided that the convenience sampling was for interview and not stool sampling

In line 125 of the original manuscript, ‘a’ has been introduced

In line 147 of the original manuscript, ‘Urine’ has been replaced with ‘urine’

In lines 457 and 462 of the original manuscript, ‘contradict’ has been replaced with ‘contrast’

**Conclusion: The reviewer recommended this section to be revised to make it more specific to the MCA model**

Sections under Conclusion that previously referred to recommendations on poverty alleviation have been removed and focus made on the MCA model and S. mansoni prevalence & intensity

**Reviewer #2: 1. The reviewer recommended that the contribution of each author be specified**

Similar symbols have been used to identify authors that had similar contribution to this manuscript.

**Reviewer #2: 2. The reviewer recommended that the last sentence under methods section of the abstract be broken down.**

The last sentence of the methods section under the abstract has been subdivided to enhance clarity and ease of understanding.

**Reviewer #2: 3. The reviewer recommended that the country in question in line 13 & 14 be made clear**

Clarification has been provided that Walukuba Division is found in Eastern Uganda

**Reviewer #2: 4. The reviewer indicated that the document had several grammatical errors that called for further review**

The manuscript has been revised to rectify typographical and grammatical errors

**Reviewer #2: 4. The reviewer indicated that the table headers reported schistosomiasis infection instead of S. mansoni infection**

The entire document has been revised to replace ‘schistosomiasis’ with ‘s. mansoni’ where applicable

We hope our revisions have adequately addressed the issues raised by reviewers and look forward to hearing from you.

Isaiah Omondi

Corresponding author
